# Effects of temperature on the behaviour and metabolism of an intertidal foraminifera and consequences for benthic ecosystem functioning

**DOI:** 10.1038/s41598-021-83311-z

**Published:** 2021-02-17

**Authors:** Noémie Deldicq, Dewi Langlet, Camille Delaeter, Grégory Beaugrand, Laurent Seuront, Vincent M. P. Bouchet

**Affiliations:** 1 Laboratoire d’Océanologie et de Géosciences, Univ. Lille, CNRS, Univ. Littoral Côte d’Opale, UMR 8187, LOG, 59000 Lille, France; 2 The Laboratory, Marine Biological Association, The CPR Survey, Citadel Hill, Plymouth, UK; 3 Department of Marine Resources and Energy, Tokyo University of Marine Science and Technology, 4-5-7 Konan, Minato-ku, Tokyo, 108-8477 Japan; 4 Department of Zoology and Entomology, Rhodes University, Grahamstown, 6140 South Africa

**Keywords:** Behavioural ecology, Climate-change ecology, Ecosystem ecology

## Abstract

Heatwaves have increased in intensity, duration and frequency over the last decades due to climate change. Intertidal species, living in a highly variable environment, are likely to be exposed to such heatwaves since they can be emerged for more than 6 h during a tidal cycle. Little is known, however, on how temperature affects species traits (e.g. locomotion and behaviour) of slow-moving organisms such as benthic foraminifera (single-celled protists), which abound in marine sediments. Here, we examine how temperature influences motion-behaviour and metabolic traits of the dominant temperate foraminifera *Haynesina germanica* by exposing individuals to usual (6, 12, 18, 24, 30 °C) and extreme (high; i.e. 32, 34, 36 °C) temperature regimes. Our results show that individuals reduced their activity by up to 80% under high temperature regimes whereas they remained active under the temperatures they usually experience in the field. When exposed to a hyper-thermic stress (i.e. 36 °C), all individuals remained burrowed and the photosynthetic activity of their sequestered chloroplasts significantly decreased. Recovery experiments subsequently revealed that individuals initially exposed to a high thermal regime partially recovered when the hyper-thermic stress ceased. *H. germanica* contribution to surface sediment reworking substantially diminished from 10 mm^3^ indiv^−1^ day^−1^ (usual temperature) to 0 mm^3^ indiv^−1^ day^−1^ when individuals were exposed to high temperature regimes (i.e. above 32 °C). Given their role in sediment reworking and organic matter remineralisation, our results suggest that heatwaves may have profound long-lasting effects on the functioning of intertidal muddy ecosystems and some key biogeochemical cycles.

## Introduction

Over the last decades, anthropogenic pressures such as industrial activity, intensive agriculture, pollution, deforestation and overfishing have altered the terrestrial and marine biosphere^1–3^. Greenhouse gas emissions have risen substantially, affecting the global climate and the frequency and magnitude of extreme weather or climatic events such as storms, floods, droughts and heatwaves^2,4–9^. Over the period 1982–2010, extremely hot days have been more frequent along 38% of the world’s coastlines^10^ and a recent study suggests that 50% of the ocean surface may suffer from a permanent marine heatwave state by the late twenty-first century^3^. Marine heatwaves, which result from the warming of both air and seawater temperature^11,12^, have caused unprecedented mass mortalities of a wide range of intertidal species such as mussels and limpets^13–17^. In the intertidal environment, sessile and slow-moving invertebrates are more likely to be exposed to extreme temperature events. Noticeably, in temperate ecosystems, surface soft-sediment temperature (i.e. within the first centimetre) can frequently reach up to 30 °C^18^ and sometimes even 40 °C at low tide^19,20^ during spring and summer. Typically, in European Atlantic mudflats, organisms can experience daily rise in sediment temperature up to 20 °C in 2 h at emersion^19^. Consequently, intertidal species are more eurytherm than their subtidal counterparts^21–23^. However, these organisms often live close to the upper limit of their thermal tolerance window, which make them also sensitive to thermal stress^21,24^. Outside their thermal range, temperature may have adverse effects on behaviour (e.g. locomotion), metabolism and reproductive strategy, which ultimately affect species survival^1,21,25^. To alleviate a thermal stress, organisms typically decrease their metabolic rate by reducing their activity such as locomotion and feeding, which decrease the space they explore and hamper their foraging strategy^21,26–28^. Thermal stress may have substantial implications for soft-bottom ecosystem functioning and services. Indeed, the movements of benthic species affect biogeochemical or ecosystem processes since they contribute to sediment reworking and dissolved material fluxes^29–33^. In this context, assessing how temperature might affect movements, activity and metabolic rate of intertidal organisms is a critical prerequisite to better understand how their contribution to ecosystem functioning may be affected by the increasing occurrence of marine heatwaves in the context of global warming.

In soft sediment, macrofaunal taxa such as molluscs, shrimps or crabs have been well-studied since they play a key role in habitat structuration^28,34–36^. Meiobenthic organisms such as benthic foraminifera also play a major role in biogeochemical or ecosystem processes^37–41^. Yet, little is known about their behavioural and metabolic response to changing temperatures. Many studies have shown that temperature can affect intertidal foraminifera survival, diversity, growth, morphology and feeding^20,42–46^ and that some foraminiferal species also increase their locomotion speed and oxygen consumption up to a point where temperature negatively impede movement, behaviour and metabolism^42,47^. Under moderate temperature, *Haynesina germanica* is the most active species (i.e. with an important time allocated to motion) amongst dominant European mudflat foraminifera and may be a key contributor to sediment reworking^48,49^. Furthermore, *H. germanica* can sequester chloroplasts from diatoms to use them for photosynthesis, which implies that this species contributes to both oxygen consumption and production in the sediment^47^. In contrast to tropical species^45,46,50,51^, the metabolic response of *H. germanica* to changing temperatures remains unknown. Given its high abundance in temperate intertidal mudflats^52–55^, high level of activity and subsequent putative contribution to sediment reworking*, H. germanica* is a good candidate to experimentally assess the effects of temperature on soft-bottom ecosystem functioning, especially in the context of global warming.

The objectives of this study are (i) to experimentally describe the responses of *H. germanica* to temperature in terms of motion behaviour and metabolic rate using a thermal gradient usually encountered in temperate intertidal environments (i.e. 6–30 °C), (ii) to characterize the effects of experimentally-induced heatwaves ranging from 32 to 36 °C and (iii) to experimentally assess the ability of the species to recover after being exposed to extreme temperatures i.e. 6 and 36 °C. We also discuss possible consequences of an acute hyperthermic stress on *H. germanica* and its putative effects on benthic ecosystem functioning and services.

## Methods

### Collection

Surface sediment (0–1 cm) were gently scrapped off with a spoon in April, May and June 2019 in two intertidal mudflats located on the French coasts of the eastern English Channel, i.e. Authie Bay (50° 22′ 20′′ N, 1° 35′ 45′′ E) and Boulogne-sur-Mer harbour (50° 43′ 6′′ N, 1° 34′ 25′′ E). Both sampling sites showed similar grain size (20% sand, 80 silt), TOC contents (between 1 and 2%)^55^, temperature and salinity values (18 °C, 33.8 PSU)^56^. Samples were stored in plastic containers (100 ml) and transported to the laboratory, then washed through a 125 µm mesh sieve. Living *H. germanica* of similar size were sorted individually with a brush and subsequently kept for 24 h in temperature-controlled incubators (MIR-154, Panasonic, Japan; temperature fluctuation ± 0.3 °C, light intensity 170 µmol m^−2^ s^−1^). Temperatures at which individuals were acclimated corresponded to those used for the experiments (i.e. 6, 12, 18, 24, 30, 32, 34 and 36 °C, see section below). Additionally, the temperature was monitored inside each incubator with a temperature logger (DSL1922L iButttons, resolution 0.1 °C, Supplementary Fig. S1). Only active individuals (i.e. producing a displacement track on a thin layer of sediment)^57–59^ were chosen and subsequently imaged to assess the shell size parameter measurements (Olympus SZX16, Japan, TC capture software with a calibrated tool for the estimation of the maximum length and width of each individual) prior to each experiment.

### Motion behaviour and recovery experiments

Active individuals were transferred into a 400 ml aquarium containing 25–30 ml of de-frozen sediment (i.e. ~ 1 cm thick) corresponding to their sampling site, free of moving animals with oxygenated overlaying natural seawater (33PSU; Supplementary Fig. S2). Eight temperatures (6, 12, 18, 24, 30, 32, 34, and 36 °C; see Supplementary Fig. S1 for temperature records) were tested. The ranges 6–30 °C and 32–36 °C were respectively considered as usual (i.e. temperature regularly experienced in the field) and extreme (i.e. temperature rarely or never reached so far in the field) temperatures in the intertidal mudflats located along the French side of the eastern English Channel. Fifteen experiments containing between 20 and 30 individuals were performed in temperature-controlled incubators (MIR-154, Panasonic, Japan, temperature fluctuation ± 0.3 °C, light intensity 170 µmol m^−2^ s^−1^) in April, May and June 2019 (Supplementary Table S1). Living foraminifera were randomly placed on the sediment surface and the displacement of each individual in and on the sediment was recorded using time-lapse photography (i.e. one image every 10 min during 24 h; Nikon V1 with a Nikkor 10–30 mm lens). Then, the images were analysed by using the software Fiji^60^. Such a method allowed us to visually follow each individual and extract the coordinates from each of the ~ 144 images combined by the computer program. The coordinates thereby gave the individual’s trajectory during the time of the experiment.

Additional recovery experiments were performed on one of each experiment carried out at 6 and 36 °C to assess specifically the resilience of *H. germanica* at extreme temperatures i.e. near the limit of their thermal range. To do so, one of each 24-h experiments carried out at 6 °C and 36 °C were pursued for extra 24-h by increasing or decreasing the temperature until 18 °C, respectively. Displacements were subsequently recorded every 10 min for 24 h. The mean distance travelled within 10 min was calculated with a 3-order simple moving average to reduce the influence of short-term fluctuations.

### Motion-traits

A total of 713 active (i.e. moving) individuals was initially selected for the experiment. During the experiment, it was not possible to track all individuals (i) because some burrowed into the sediment up to a depth where their paths were not visible and/or (ii) because some paths crossed and consequently individual trajectories were lost. We therefore only kept individuals that exhibited visible tracks throughout the whole 24 h experiment. In total we followed the trajectories of 246 individuals.

Four motion traits were investigated following Seuront and Bouchet^49^ and Deldicq et al.^48^.

First, the level of activity (i.e. time allocated to locomotion by each individual) was estimated with the activity index *A*_*i*_ which is based on the ratio *t*_move_ and *t*_active_ as follows:

$$A_{i} = { 1}00 \times \left( {t_{{{\text{active}}}} /t_{{{\text{move}}}} } \right).$$

where *t*_*move*_ includes the total time taken by an individual to move from its initial to its final position, which thereby includes the time periods when individual remains inactive. In contrast, *t*_*active*_ only considers the time periods when an individual actually moves between its initial and final position.

The distance travelled by each individual between two images (i.e. 10 min) was assessed as follows:

$$D_{t} = \, \surd \left( {\left( {x_{t} {-}x_{t+1} } \right)^{{2}} + \, \left( {y_{t} {-}y_{t+1} } \right)^{{2}} } \right).$$

where (*x*_*t*_,*y*_*t*_) and (*x*_*t*+1_, *y*_*t*+1_) are the coordinates between two successive images taken at times *t* and *t* + 10 min and the total distance travelled within 24 h was then calculated (*D*_24_) and normalized by the experiment duration to obtain velocity.

The complexity of the trajectory of each individual was assessed using fractal dimension analysis. Because the principles behind fractal theory, fractal analysis techniques and their applications to behavioural data, including foraminifera behaviour^48^, have all been described in detail elsewhere^61–63^, we only briefly describe hereafter the basic principles of the box-counting method, which is likely among the most widely applied and intuitive methods available to date to characterize the geometric complexity of movement paths. This method superimposes a regular grid of squares of length *l* on a path and counts the number of occupied squares, *N*(*l*). This procedure is repeated using different values of *l*. The surface occupied by a trajectory is then estimated using a series of boxes spanning a range of surfaces down to some small fraction of the entire space, typically the size of the organism considered. The number of occupied squares fundamentally increases with decreasing square size, and the presence of a fractal structure manifests itself by a power–law relationship of the form *N*(*l*) = *k* × *l*^−*D*^, where *k* is an empirical constant and *D* the fractal dimension. The fractal dimension *D*, estimated from the slope of the linear trend of the log–log plot of *N*(*l*) versus *l*, fundamentally measures the degree to which the trajectory fills the available space and is bounded between *D* = 1 for a line (i.e. the simplest instance of a trajectory) and *D* = 2 for a movement so complex that it actually fills the whole available space.

**Figure 1:**
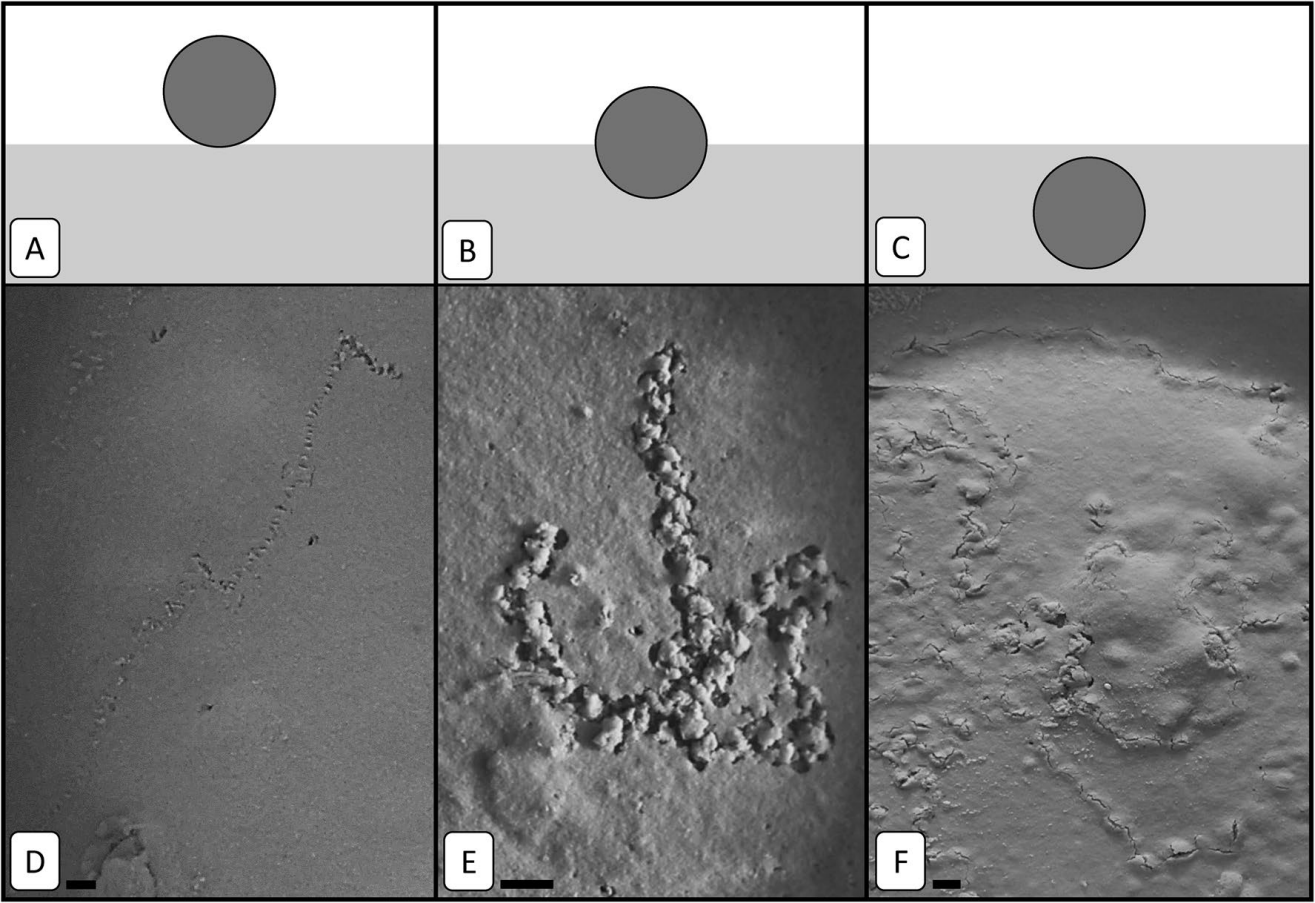
Schematic side-view representation of the vertical position (**A**–**C**) and top-view images of the sediment surface showing actual trajectories of foraminifera (**D**–**F**) related to the three vertical position categories, which can be taken by a foraminifera, i.e. surface (**A**,**D**) sediment–water interface, (**B**,**E**), and burrowed (**C**,**F**). Scale bars = 0.2 mm. From Deldicq et al.^48^.

Following the method newly described in Deldicq et al.^48^, the vertical position of *H. germanica* in the sediment for every individual and picture was determined based on a classification with three depth categories. When part of the test remained visible at the surface and the width of the path was indistinguishable an individual was considered to be crawling on the sediment surface (Fig. 1A,D). When an individual was burrowing into the sediment, its position was divided into two categories: it was considered (i) as moving at the sediment–water interface when half of the test was visible (Fig. 1B,E) and (ii) as fully burrowed into the sediment when a swelling at the sediment surface was the only indication of the presence of a test in the sediment (Fig. 1C,F). The number of individuals was estimated for each position and each 10-min period during the time of the experiment.

### Surface sediment reworking rate

To assess *H. germanica* contribution to surface sediment reworking, the test surface *TS*_*i*_ (mm^2^) of each individual was estimated by measuring individual maximum length and width and assuming that the species has an ellipse-shape shell:

$${TS}_{i}=\uppi \times \frac{\mathrm{Length}}{2} \times \frac{\mathrm{Width}}{2}.$$

Since there was no significant difference in term of individual size between each set of experiment (Kruskal–Wallis test, *p* < 0.05), the mean test surface *TS* was calculated for each set of experiment and used for the calculation of the Individual Surface Sediment Reworking Rate, *SSRR*_*i*_ (mm^3^ indiv^−1^ day^−1^):

$$SSRR_{i} = TS \times D_{{{24}}} .$$

where *D*_24_ is the total distance travelled (in mm) within 24h by each individual.

### Oxygen consumption and production

Active individuals used for respiration measurements were acclimated overnight with artificial seawater (35 g of Red Sea salt per litter of MilliQ ultrapure water, and referred to as ASW hereafter) at the temperature corresponding to the experimental condition (i.e. 6, 12, 18, 24, 30 and 36 °C). Three sets of five active individuals (with homogenised shell length ranging from 340 to 420 µm, Kruskal–Wallis test, *p* < 0.05) were transferred to a 1-mm wide and 1-cm high glass microtube containing ASW for each chosen temperature (6, 12, 18, 24, 30 and 36 °C, Supplementary Table S2). Measurements within the microtube were carried out in a temperature-controlled water bath (Huber CC-K12, Germany) to estimate oxygen fluxes at 6, 12, 18, 24 and 30 °C. To this end, a 50-µm Clark-type oxygen microelectrode (Unisense, Denmark) was 2-point calibrated^64^ using oxygen-saturated seawater (considering O_2_ saturation at 35 PSU and at the chosen temperatures) and an anoxic solution (20 g of sodium ascorbate per litter of 0.1 mol l^−1^ NaOH solution). The electrode was then placed in the measurement microtube about 300 µm above the 5 individuals. Oxygen profiles were realized with a 50-µm vertical resolution to determine the oxygen consumption gradient (d*C*/d*z*, in pmol cm^−4^) in the first millimetre above the foraminifera^38,59^.

Oxygen consumption gradients were first measured in the dark to estimate foraminiferal respiration and then oxygen production gradients were estimated under homogeneous light conditions to determine net photosynthesis (photosynthetically active radiation 170 µmol photon m^−2^ s^−1^; SA-190 quantum sensor, LI-COR, USA, provided by two arrays of LEDs (YN-160 III, Yongnuo, China). Given that previous studies show that ASW alone does not produce nor consume oxygen^59,65,66^, no further blank controls were performed for this experiment and the measured oxygen production of consumption was assumed to originate from the foraminifera themselves.

### Respiration and photosynthesis calculations

Oxygen fluxes *J* (pmolO_2_ cm^−1^ s^−1^) were calculated using Fick’s first law of free diffusion, as follows:

$$J = D \times dC/dz,$$

where *D* is the free diffusion coefficient for oxygen in seawater at a given temperature^49^ and *dC/dz* the oxygen gradient 1 mm above the foraminifera in the microtube. Oxygen solubility and free diffusion coefficients (*D*) were selected from tables compiled by Ramsing and Gundersen^67^ (Unisense, Denmark). All respiration measurements were performed in the dark in a temperature-controlled water bath (Huber CC-K12, Germany).

Individual respiration rate *R* (pmolO_2_ indiv^−1^ h^−1^) and net photosynthesis rate *NP* (pmolO_2_ indiv^−1^ h^−1^) were subsequently calculated as:

$$R = J_{dark} \times S/n,$$

$$NP \, = \, J_{light} \times S/n,$$

where *S* is the microtube inner section (*S* = 7.9 × 10^–3^ cm^2^), *n* the number of individuals (i.e*. n* = 5) and *J* the fluxes estimated under dark and light conditions, respectively.

Gross photosynthesis (*GP*) was estimated from respiration (*R*) and net photosynthesis (*NP*) rates as follow:

$$GP \, = \, NP \, + \, R.$$

In addition, to estimate the influence of temperature on *H. germanica* physiological rate, Q_10_ was calculated within the ranges 6–24 °C and 24–36 °C. The Q_10_ values quantify changes in the metabolic rate for a 10 °C increase:

$${\text{Q}}_{{10}} = \left( {\frac{{R(T_{2} )}}{{R(T_{1} )}}} \right)^{{\frac{{10}}{{T_{2} - T_{1} }}}} .$$

where *R*(*T*_1_) and *R*(*T*_2_) (nmolO_2_ indiv^−1^ h^−1^) are the metabolic rate (i.e. respiration or gross photosynthesis) respectively measured at extreme tested temperatures (i.e. 6 and 36 °C) and 24 °C.

To estimate the daily oxygen budget, i.e*.* the balance between oxygen consumption (respiration) and production (photosynthesis) within a day, we calculated the amount of oxygen produced in a day for a 12-h light exposure duration (to account for diurnal cycles) and 6-h light exposure duration (to account for both diurnal and tidal cycles, assuming that coastal seawater turbidity is so high that no light is reaching the sediment during immersion). Such calculations were done by pondering net photosynthesis with respiration rates with a 0.5 and 0.75 ratio for 12-h and 6-h light exposure, respectively.

### Data analysis

Because behavioural parameters were non-normally distributed (Shapiro–Wilk test, *p* < 0.05). Kruskal–Wallis tests were conducted for activity and surface sediment reworking rate in order to discriminate temperatures. In case of significant differences a Dunn post-hoc test was applied for two-sample comparisons^68^. In turn, metabolic parameters rate were normally distributed (Shapiro–Wilk test, *p* > 0.05) and an analysis of variance (ANOVA) was conducted on respiration rates and photosynthesis followed by a two-sample comparison (Tukey test) to identify distinct groups of measurement^68^. The presence of significant differences between fractal dimensions was assessed using an analysis of covariance. All statistical analyses were performed using R.3.5.2. software^69^.

## Results

### Motion traits

**Figure 2:**
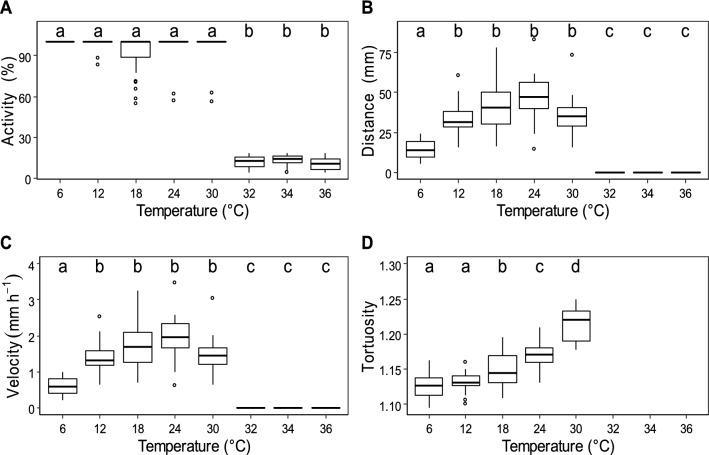
The influence of temperature on (**A**) the activity (**B**) the distance travelled (over 24 h), (**C**) the velocity and (**D**) the fractal dimension of *H. germanica*. The box represents the first, second and third quartiles and the whiskers extend to 1.5 times the interquartile range; values outside this range are represented by open circles. Number of replicates are 30, 23, 30, 25, 15, 69, 28 and 26 for 6, 12, 18, 24, 30, 32, 34 and 36 °C respectively. Due to the absence of motion it was impossible to estimate fractal dimension at 32, 34 and 36 °C. Letters above the boxes (‘a’, ‘b’, ‘c’ and ‘d’) identify significant different groups (Dunn test, *p* < 0.05).

Individuals were most active between 6 and 30 °C, spending more than 90% of their time moving into the sediment (Fig. 2A). Individuals exposed to extremely high temperatures (i.e. 32–36 °C) significantly decreased their activity from *circa* 90% to *ca.* 15% (Dunn test, *p* < 0.01; Fig. 2A).

The highest velocities and the longest distances travelled during the 24-h experiment were observed in the range 12–30 °C (Fig. 2B,C). The longest trajectories were measured at 24 °C with a mean travelled distance of 46.9 mm (Fig. 2B,C). Beyond 32 °C, individuals started burrowing into the sediment at the beginning of the experiment but there was no subsequent displacement throughout the rest of the experiment (Fig. 2B,C). More specifically, the travelled distance of *H. germanica* trajectories were discriminated into several groups, i.e*. D*_*t*(36 °C)_ = *D*_*t*(34 °C)_ = *D*_*t*(32 °C)_ < *D*_*t*(6 °C)_ < *D*_*t*(12 °C)_ = *D*_*t*(18 °C)_ = *D*_*t*(24 °C)_ = *D*_*t*(30 °C)_ (Dunn test, *p* < 0.01).

Since there were no displacements between 32 and 36 °C, the complexity of movement (i.e. fractal analysis) was not assessed for these temperatures. However, all trajectories considered at cooler temperature (i.e. 6, 12, 18, 24, 30 °C) were characterized by a fractal property, i.e. a highly significantly linear behaviour of *N*(*l*) vs. *l* in log–log plots (*r*^2^ > 0.99, *p* < 0.01). The fractal dimension *D* ranged from 1.09 to 1.22 and significantly differed between treatments (Fig. 2D; Kruskal–Wallis test, *p* < 0.01). The trajectories of *H. germanica* was subsequently discriminated into several homogeneous groups, i.e.* D*_6 °C_ = *D*_12 °C_ < *D*_18 °C_ < *D*_24 °C_ < *D*_30 °C_, which overall indicated an increase in movement complexity with rising temperature.

**Figure 3:**
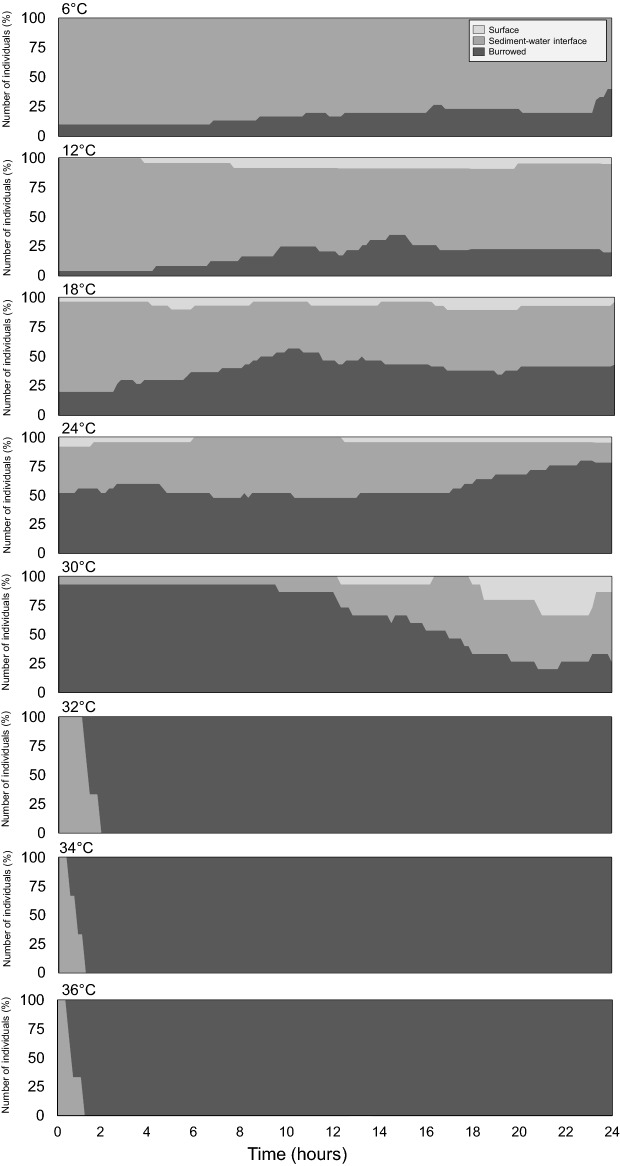
Temporal changes in the vertical position of *H. germanica* for each tested temperature. Number of individuals are shown in Supplementary Table S1.

For intermediate temperatures (18, 24, 30 °C), individuals were alternatively observed at the sediment–water interface or burrowed in the sediment during the experiment (Fig. 3). At the hottest temperatures e.g*.* 32–36 °C, individuals moved rapidly from the surface down to the sub-surface and stayed buried during the remaining time of the experiment. In contrast, they were observed at the sediment–water interface between 6 and 12 °C (Fig. 3).

### Recovery experiment

**Figure 4:**
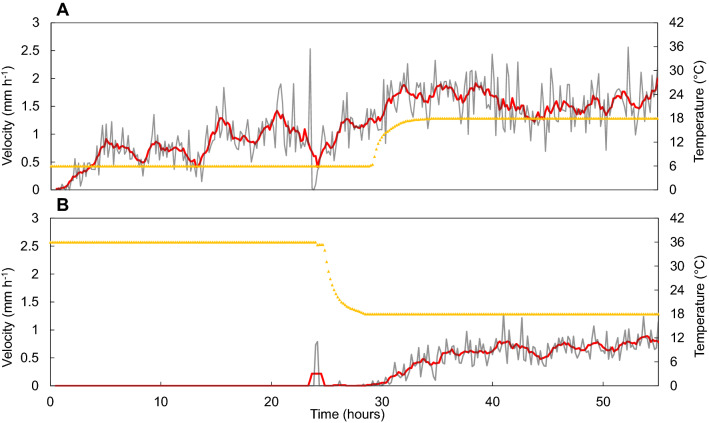
Temporal changes in the mean velocity of 9 *H. germanica* individuals previously exposed at (**A**) 6 °C then 18 °C and (**B**) 36 °C then 18 °C. The grey line is the instantaneous velocity and the red line is the 3-order simple moving average of the velocity. Yellow triangles correspond to water-temperature changes through time.

After a 24-h exposure to a temperature of 6 °C, individuals exposed to 18 °C increased their velocity from an average of 0.8 mm h^−1^ in the first 24 h of the experiment up to approximately 1.6 mm h^−1^ in average over the 30–55 h time interval (Fig. 4A). This increase started as soon as the temperature rose in the experiment container (Fig. 4A). Noticeably, the recovered velocity at 18 °C (1.6 mm h^−1^) was close to the value observed for individuals solely exposed to 18 °C (1.74 mm h^−1^, Fig. 2C).

At 36 °C, the distance travelled was nearly nil during the first day of the experiment. Individuals exposed to 36 °C for a 24-h period started to move only 4 h after the decrease in temperature from 36 to 18 °C (Fig. 4B). The recovered mean velocity at 18 °C (0.57 mm h^−1^) never reached the mean velocity where individuals were solely exposed to a thermal regime of 18 °C (1.7 mm h^−1^; Fig. 2C).

### Respiration and photosynthesis

**Figure 5:**
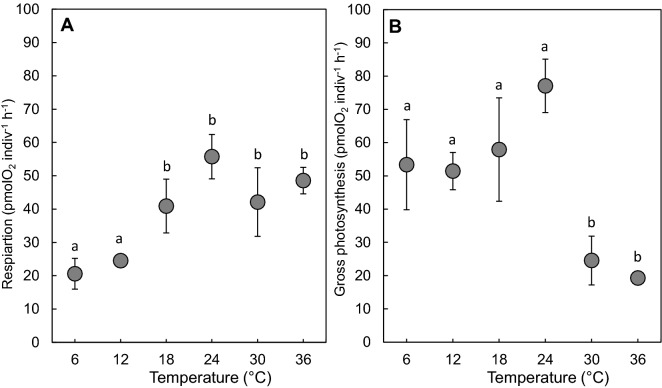
Mean values of (**A**) respiration and (**B**) gross photosynthesis (pmolO_2_ indiv^−1^ h^−1^) of *H. germanica* under different thermal regime in 3 replicate measurements. The error bars are the standard errors of the mean. Letters ‘a’ and ‘b’ identify significant different groups (Tukey test, *p* < 0.05).

Oxygen respiration rates did not significantly differ between 6 and 12 °C (Tukey test, *p* < 0.01). However, respiration rates were significantly higher for warmer temperatures (Tukey test, *p* < 0.01). Hence, oxygen consumption increased from 24.5 pmolO_2_ indiv^−1^ h^−1^ (12 °C) to 55.7 pmolO_2_ indiv^−1^ h^−1^ (24 °C), then decreased down to 48.5 pmolO_2_ indiv^−1^ h^−1^ at 36 °C (Fig. 5A). Gross photosynthesis also increased up to 77 pmolO_2_ indiv^−1^ h^−1^ when temperature warmed from 6 to 24 °C. A significant diminution was subsequently observed from 24 °C to 30 °C (Tukey test, *p* < 0.01; Fig. 5B).

The increase in respiration and gross photosynthesis between 6 and 24 °C can be described with Q_10_ = 1.75 and Q_10_ = 1.22, respectively. However, the influence of the warmest temperatures on respiration decrease (Q_10_ = 0.89) was lower than for gross photosynthesis decrease (Q_10_ = 0.32) over the 24–36 °C range.

### Surface sediment reworking rate and oxygen budget

**Figure 6:**
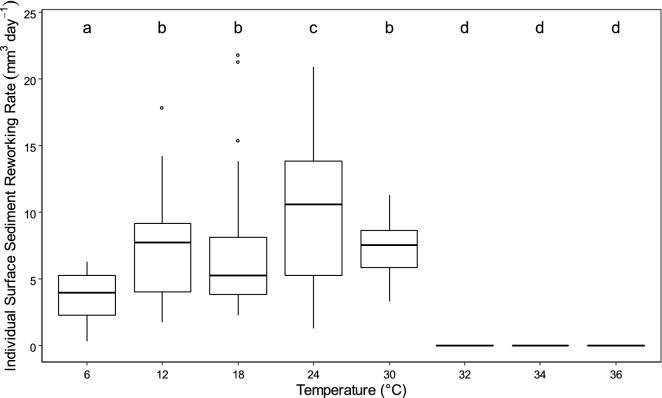
Individual surface sediment reworking (mm^3^ indiv^−1^ day^−1^) of *H. germanica* under different thermal regime. Letters above plots (‘a’, ‘b’, ‘c’) indicate significant differences among measurements (Dunn test, *p* < 0.05). The box represents the first, second and third quartiles and the whiskers extend to 1.5 times the interquartile range; values outside this range are represented by open circles.

Due to low travelled distances, there was no surface sediment reworking beyond 32 °C. In contrast, for lower temperatures, individuals could rework between 3.7 and 10.1 mm^3^ indiv^−1^ day^−1^ (respectively 6 and 24 °C; Fig. 6). Statistical analyses showed significant differences in the *SSRR*_*i*_ between temperatures (Kruskal–Wallis test, *p* < 0.05) and four groups were further identified as *SSRR*_*i*(32 °C)_ = *SSRR*_*i*(34 °C)_ = *SSRR*_*i*(36 °C)_ < *SSRR*_*i*(6 °C*)*_ < *SSRR*_*i*(12 °C)_ = *SSRR*_*i*(18 °C)_ = *SSRR*_*i*(30 °C)_ < *SSRR*_*i*(24 °C)._ Q_10_ of surface sediment reworking in the thermal range 6–24 °C was 1.75.

**Figure 7:**
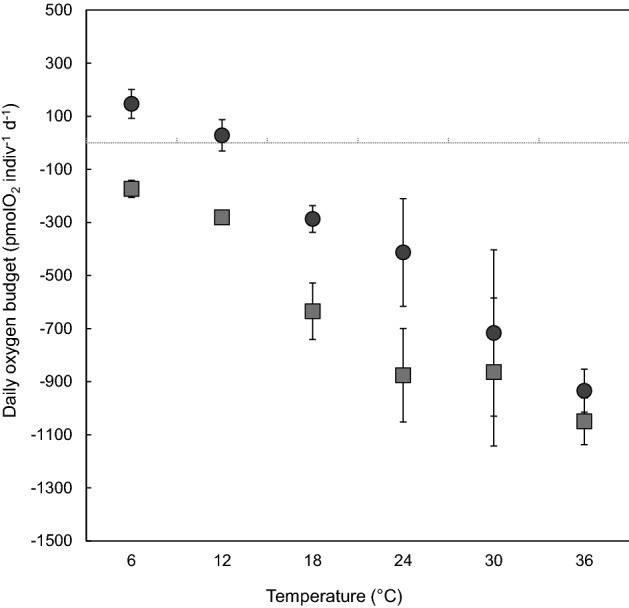
Daily oxygen budget of *H. germanica* (pmolO_2_ indiv^−1^ day^−1^) under 12 h (black dots) and 6 h (grey squares) light exposure and thermal regimes. The error bars are the standard errors calculated on the 3 replicates at each temperature.

For a 6-h light exposure, daily oxygen budget was negative at all temperatures and significantly decreased above 12 °C (Fig. 7, Tukey test *p* < 0.05). When considering a 12 h light exposure cycle, average daily oxygen productive was positive at 6 and 12 °C and gradually decreased to reach negative values within the thermal range 18–36 °C.

## Discussion

### The resilience of *H. germanica* motion behaviour to temperature fluctuations reveals plasticity to seasonal thermal variations

*Haynesina germanica* was more active in the range 6–30 °C, with the highest velocities and distances travelled being in the range 12–24 °C. Specifically, individuals were 1.4 times faster at 24 °C than at 12 °C (Fig. 2C). This is consistent with previous measurements of locomotion speed on glass petri dish, velocity being nearly twice lower at 12 °C (~ 2 mm h^−1^)^57^ than at 22 °C (~ 4 mm h^−1^)^70^. This observation confirms that cold temperatures may reduce the activity of temperate foraminifera^42^. In our experiments, *H. germanica* explored actively its environment from 6 to 30 °C by consistently moving vertically and horizontally into the sediment between 22 and 24 h. However, specimens remained only active between 3 and 4 h in the sediment at temperatures above 30 °C with velocities and travelled distances being nil above 32 °C. Increasing fractal dimensions in the range 12–30 °C were also indicative of more intensive foraging behaviour consistent with the more complex trajectories and more intensive foraging behaviour exhibited by unstressed organisms^49,71–73^. Note that these results may also indicate that foraging behaviour may differ at the sediment–water interface and within the sediment. The observed adaptive responses to a range of temperatures typically encountered in temperate intertidal mudflats (i.e. 6–30 °C^19,74,75^) as well as more extreme and rare temperature (36 °C) suggest that *H. germanica* behavioural flexibility specifically evolved to optimize the timing of their response to thermal stress at temporal scales typical of the tidal alternance of immersion and emersion. In fact, many studies have shown that intertidal invertebrates often live close to the upper limit of their thermal tolerance windows^24,76–78^. Our findings therefore suggest that irrespective of species physiological and behavioural plasticity, unusual temperatures such as those caused by heatwaves may affect species performance and perhaps survival. After being exposed to extremely hot temperatures, *H. germanica* was nevertheless able to quickly recover. After bringing them back to 18 °C, all individual exposed to cold and hot temperatures (6 °C and 36 °C), started exploring all potential habitats i.e. both surface and deeper sediment, suggesting that the protist can exhibit a thermotactic behaviour.

### Thermal control of the position of *H. germanica* in the sediment

At temperatures corresponding to autumn and winter (i.e. 6–12 °C), *H. germanica* preferably remained at the sediment–water interface. At intermediate temperatures (18 and 24 °C) corresponding to spring and summer conditions, individuals alternatively moved in and on the sediment during the whole experiment with a proportion of burrowed individuals increasing with temperatures. For instance, at 30 °C more than 90% of the individuals were observed below the sediment–water interface. Habitat selection as a function of environmental conditions has also been reported in a wide range of organisms such as crabs, worms and gastropods^28,79,80^. Organisms inhabiting intertidal mudflats move toward a more favourable habitat following the vertical thermal gradient they experience in soft sediments^81,82^. Under low temperatures (here 6, 12 °C), basking behaviour, i.e. a common thermoregulatory behaviour observed in many ectotherms, might allow species to live in the limited-oxygenated zone to draw benefit from solar heating^83–87^. In contrast, burrowing deep into the sediment may provide cooler environment and leads to a decrease in cell temperature^79,88,89^. Considering that the thin sediment layer used in our experiments is unlikely to generate a thermal gradient, our results strongly suggest that benthic foraminifera, in particular *H. germanica*, may have an intrinsically basking- and burrowing behaviour to regulate their inner body temperature.

### Effect of temperature on *H. germanica* metabolism: an adaptation to variable thermal forcing

In our experiments, highest respiration and photosynthesis rates were recorded between 18 and 24 °C. Outside this range, *H. germanica* respiration rates strongly decreased at cooler temperatures (6, 12 °C) while there was a decrease in gross photosynthesis at 30 °C. Metabolic change is a common response to temperature in ectothermic species^90^, including benthic and planktonic foraminifera^42,91^. Instability in metabolism affects macro-invertebrate species performance such as feeding, mating and locomotion^92–95^, which is consistent with our observations on *H. germanica* motion-behaviour, where travelled distances, and hence velocities, consistently decreased at cooler and warmer temperatures. Our results open a new perspective on our understanding of the physiology of *H. germanica*. In our experiments, the Q_10_ values reported in the range 6–24 °C for respiration (Q_10_ = 1.75) and photosynthesis (Q_10_ = 1.22) suggest (i) a maximum performance level and a relatively low thermal dependence of respiration and (ii) that photosynthesis is not affected by temperature inside this thermal range. Low Q_10_ values have been interpreted as characteristic of the optimal temperature range of a species in its natural habitat^96^. Noticeably, our Q_10_ calculated on respiration is substantially lower than previous direct Q_10_ estimates for planktonic foraminifera^91^ (Q_10_ = 3.18) and for the intertidal foraminifera *Ammonia beccarii tepida*^42^ (Q_10_ = 3.2 in the north-eastern regions of the Pacific) but in the same order of magnitude as Arcachon Basin mudflats for *Ammonia tepida* and *Haynesina germanica*^58^ (Q_10_ = 1.4 and Q_10_ = 1.8 respectively). Compared to other meiobenthic species from the English Channel mudflats, *H. germanica* respiration Q_10_ in the 6–24 °C range is lower than those reported in the 0–20 °C range in the sabellid polychaete *Manayunkia aestuarina* (Q_10_ = 2.19) and in the copepod *Tachidius discipes* (Q_10_ = 2.17)^97^. Our findings suggest that the protist is particularly well adapted to the frequently-occurring thermal range 6–24 °C in intertidal soft-sediments in temperate environments. Similarly, a vast majority of intertidal macro-invertebrates can easily tolerate thermal variation with no adverse effects on their physiological rates^76,98^, like on metabolic rates of fiddler crabs^99^.

### Fast behavioural and metabolic responses of *H. germanica* to extreme temperatures: a key for survival in an era of climate change?

At high temperatures (32, 34 and 36 °C), *H. germanica* individuals immediately burrowed in the sediment and then remained inactive throughout the rest of the experiment. These two successive behaviours (i.e. burrowing then inactivity) are typically observed in macro-invertebrate intertidal species exposed to temperatures outside their tolerance thermal range^79,89,100^. Note that this strategy may also be detrimental given the low oxygen penetration depth and the intense hydrogen sulphide production in coastal marine sediments^101,102^, which are known to hamper benthic foraminifera^103–105^. Noticeably, the lethal limit of *H. germanica* was never reached since after being inactive for 24-h at 36 °C, all individuals started to move (though they never recovered their baseline behaviour and activity during the time of the experiment) when temperature decreased at 18 °C. The distance travelled at 18 °C by individuals previously exposed at 36 °C was twice lower than the distance travelled by individuals previously exposed to 6 °C, suggesting that although not lethal, the 24 h spent by *H. germanica* individuals at 36 °C had long-lasting harmful consequences. In the literature, temperature LT_50_ (i.e. the temperature for which 50% of individuals die) for intertidal foraminifera typically ranged from 37.5 to 45 °C^42^. Exposure to high temperatures have important adverse effects such as production of reactive oxygen species and DNA degradation^26,76^. These is confirmed by the metabolic Q_10_ value, which dropped below 1 in the range 24–36 °C (respectively Q_10_ = 0.89 and Q_10_ = 0.32 for respiration and gross photosynthesis), suggesting that biological functions are altered in *H. germanica* above 24 °C. Our respiration Q_10_ is similar to the one of the intertidal nematode *Pellioditis marina* from the south-western regions of the Netherlands^106^ (Q_10_ = 0.76 in the range 25–35 °C), although thermal dependence is much higher in *Ammonia beccarii tepida* from the eastern Pacific (Q_10_ = 0.17 in the 34–45 °C range)^42^ suggesting that *H. germanica* respiration might also be inhibited beyond 36 °C. Photosynthetic activity of *H. germanica* is more affected than respiration, a result that has been found in other symbiont-bearing benthic foraminifera^50,51,107^. Our results therefore suggest that *H. germanica* may not benefit from autotrophic nutrition since sequestered chloroplast photosynthetic activity was strongly inhibited beyond 24 °C. Further analyses are needed to identify whether the plastids could recover after being exposed to high temperatures and whether individuals maintain them in their cell or use them as a source of food.

### Consequences of marine heatwaves on *H. germanica* contribution to benthic ecosystem functioning and services

The shifts in metabolism and motion behaviour observed in this study provide evidence that heatwaves may alter the contribution of *H. germanica* to benthic ecosystem functioning. Specifically, sediment reworking directly depends on motion-behaviour (e.g. crawling, burrowing), which leads to sediment particle displacements^36,108^. The Q_10_ value reported in the range 6–24 °C for surface sediment reworking rate (Q_10_ = 1.75) indicated a thermal dependence in the range 6–24 °C. Hence, *H. germanica* can rework a larger amount of sediment within the range 18–30 °C. In addition, individuals intensively explored the environment by moving vertically and horizontally into the sediment. This diversity of movements would most likely lead to more intense sediment mixing since particles are carried out in both directions. In contrast, at lower temperatures, *H. germanica* remained in the upper millimetres of sediment inducing a space-scale limited contribution to surface sediment reworking. The intertidal polychaete species *Neanthes virens* also showed a lower bioturbation rate at 6 °C, which limits sediment transport and dissolved fluxes^109^. At temperatures > 32 °C, *H. germanica* surface sediment reworking activity fully ceased. Such temperatures can be reached during summer in temperate intertidal mudflats^18,19,110^. Heatwaves may therefore limit *H. germanica* contribution to surface sediment reworking. Although heatwaves have limited duration, they actually continue to increase in frequency and intensity^3^. The repetition of such extreme events over successive periods has dramatic consequences on species’ survival and associated ecosystem functions^1,6,14,17^. As previously evidenced for macro-invertebrates inhabiting the Eastern English Channel coastlines^17^, we suggest that the thermal tolerance of *H. germanica* and therefore its contribution to ecosystem functions could be altered by the successive exposition to extreme temperatures. It would be interesting to perform successive thermal exposures to high temperatures (i.e. chronic stress) to further investigate the ability of *H. germanica* to acclimate and assess its resistance and resilience to several expositions to extreme temperatures.

Benthic foraminifera may also affect benthic fluxes directly by consuming or producing oxygen. Our results suggest that foraminiferal oxygen uptake increases in the 6–24 °C range and that high temperatures may most likely limit the contribution of *H. germanica* to oxygen fluxes. Noticeably, oxygen production by photosynthesis, and to a lesser extent oxygen consumption, decreased at 30 °C and above. It further co-occurred with individuals reduced-surface sediment reworking activity during heatwaves. Our daily oxygen budget calculations under realistic light exposure revealed that *H. germanica* oxygen production was closed to 0 or negative at all measured temperatures. Specimens from Atlantic mudflats showed similar negative oxygen production under 12 h light exposure (i.e. − 283 at 13 °C and − 327 pmolO_2_ indiv^−1^ day^−1^ at 18 °C; recalculated respectively from Jauffrais et al.^111^ and Cesbron et al.^58^). Within European waters kleptoplastic intertidal species, only *Cribroelphidium williamsoni* showed positive oxygen production budget under a 12 h dark–light cycle (5165 pmolO_2_ indiv^−1^ day^−1^; recalculated from Jauffrais et al.^112^). This result confirms that *H. germanica* has a minimal impact on benthic oxygen production (up to 0.2%).

## Conclusion

Global climate change has now unambiguous effects on many marine biological and ecological systems of the world. Among observed consequences of global climate change, marine heatwaves have become more frequent and prominent. In this context, we have examined some biological responses of the temperate foraminifera *H. germanica* to thermal changes in soft-sediment habitats over a short period. Although some thermal plasticity is observed for temperatures commonly observed in the field, we show that a hyper-thermic stresses typical of a marine heatwave strongly affects the behaviour and the metabolism of this protist, triggering responses that were not entirely reversed during the time of the experiments. Our results also suggest that these biological alterations have consequences on the species contribution to surface sediment reworking.

## Supplementary Information

Supplementary Information.
