# Supplementary material for: Effects of temperature on the behaviour and metabolism of an intertidal foraminifera and consequences for benthic ecosystem functioning
Source: Sci Rep. 2021 Feb 17;11:4013. doi: 10.1038/s41598-021-83311-z (PMC7889916; doi:10.1038/s41598-021-83311-z)
Supplement: Supplementary file 1 — Supplementary Information. [file 41598_2021_83311_MOESM1_ESM.pdf]

Effects of temperature on the behaviour and metabolism of an intertidal foraminifera and consequences for benthic ecosystem functioning

Noémie Deldicq[1*], Dewi Langlet[1], Camille Delaeter[1], Grégory Beaugrand[1,2], Laurent Seuront[1,3,4], Vincent M.P. Bouchet[1]

[1]Univ. Lille, CNRS, Univ. Littoral Côte d'Opale, UMR 8187, LOG, Laboratoire d'Océanologie et de Géosciences, F-59000, Lille, France.

[2]Marine Biological Association, The CPR survey, The Laboratory, Citadel Hill, Plymouth, United Kingdom.

[3]Department of Marine Resources and Energy, Tokyo University of Marine Science and Technology, 4-5-7 Konan, Minato-ku, Tokyo 108-8477, Japan.

[4]Department of Zoology and Entomology, Rhodes University, Grahamstown, 6140, South Africa.

*Corresponding author: E-mail: noemie.deldicq@outlook.fr

Supplementary information

Supplementary figure 1. Temperatures in the incubator for each temperature tested in this study (6, 12, 18, 24, 30, 32, 34 and 36°) recorded with a temperature logger every 10 min (black curves; DSL1922L iButtons, resolution 0.1°C). Red dots corresponds to the temperature displayed by the incubator' screen every hour. Yellow line corresponds to the change of light regime from light to dark condition.

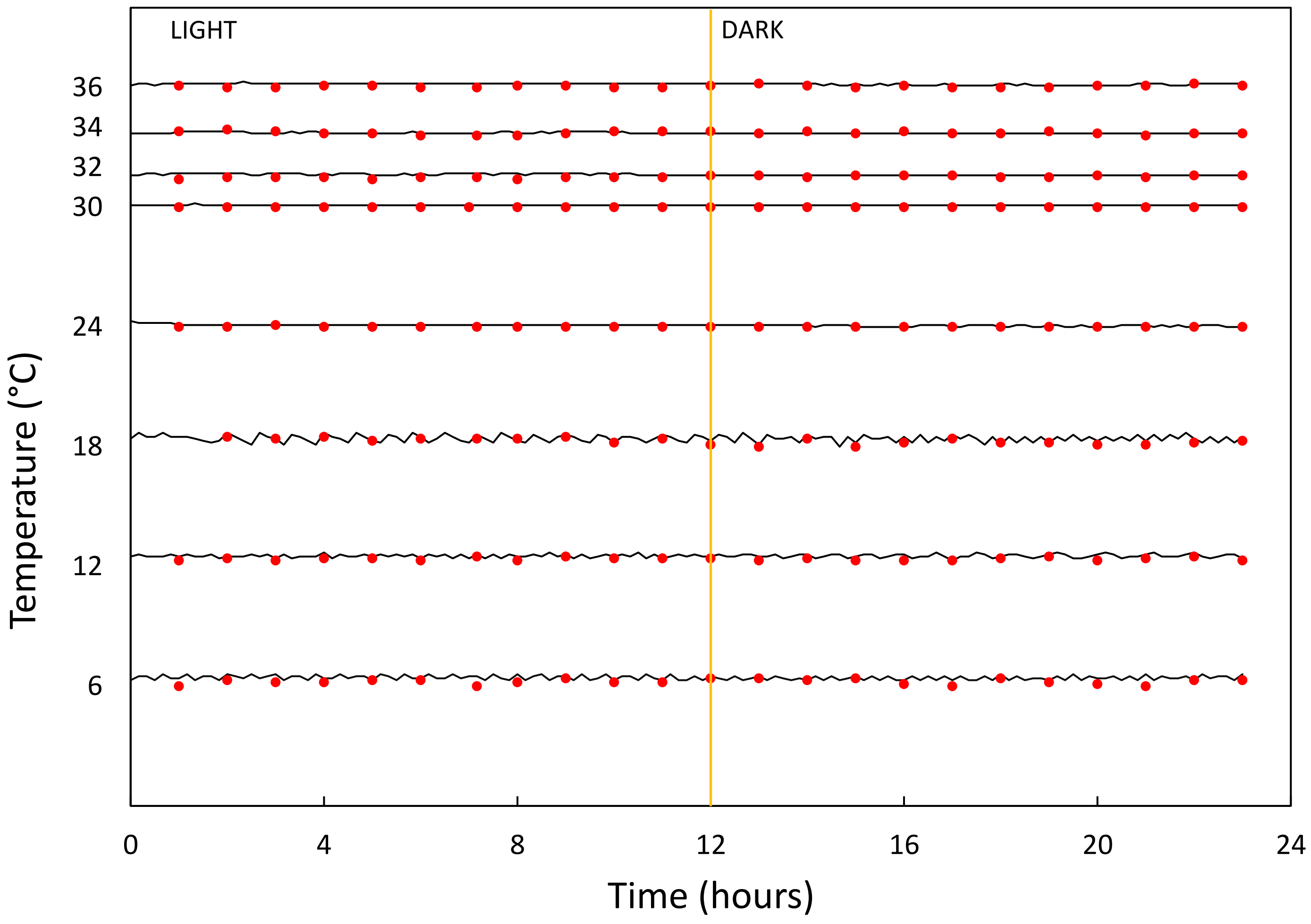

Supplementary figure 2. Experimental set-up for time-lapse assessment of foraminiferal life trait.

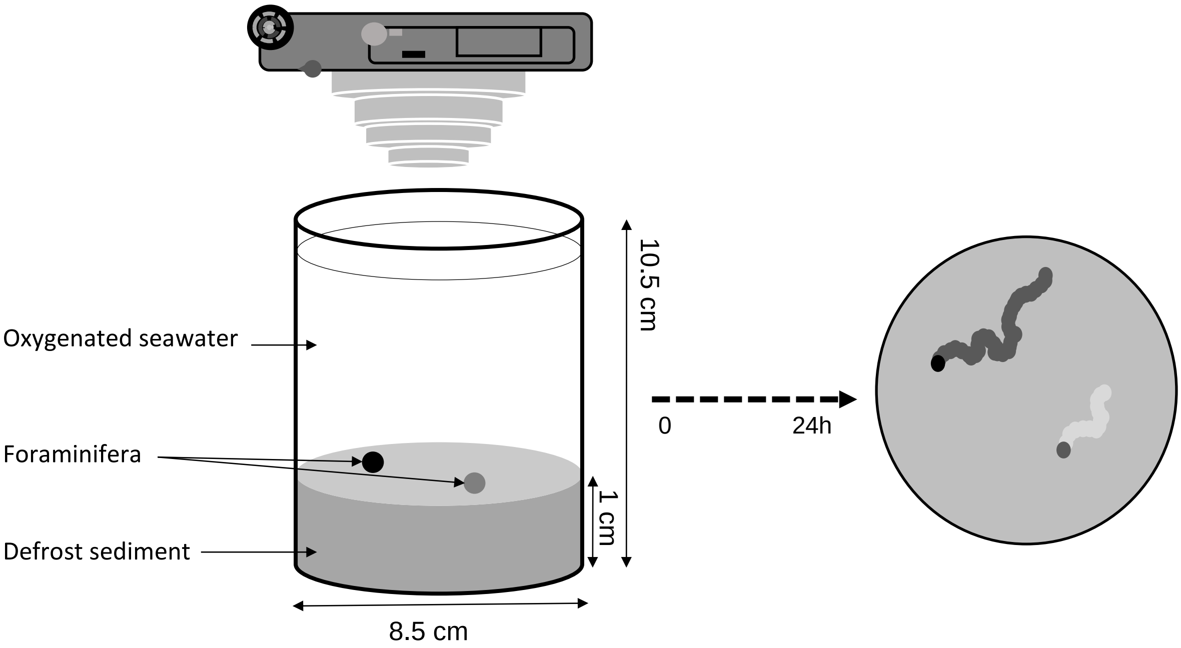

Supplementary Table 1. Date of experiment and number of individuals of *Haynesina germanica* used for different thermal regimes. N=used individuals, n=analysed individuals.

Temperature (°C)	Date of experiment (2019)	N	n
6	April 3,16,17	75	30
12	April 3,11,12,16,17	110	24
18	April 9,12,17<br>June 6,26	97	30
24	April 19,24<br>June 6,7	78	25
30	April 19,24<br>June 6	80	15
32	April 26<br>May 3,15<br>June 12,20	122	69
34	May 15<br>June 12,20	75	28
36	April 19<br>May 3<br>June 6	76	26

Supplementary Table 2. Respiration, rate, net and gross photosynthesis values of *Haynesina germanica* under different thermal regimes. N= used individuals. n= number of replicates. $\bar{X}$ denotes the mean and SE the standard error.

**Temperature (°C)**	**Date of experiments (2019)**	**N**	**n**	**Respiration rate** ($pmolO_2$ $indiv^{-1}$ h^{-1})		**Net photosynthesis** ($pmolO_2$ $indiv^{-1}$ h^{-1})		**Gross photosynthesis** ($pmolO_2$ $indiv^{-1}$ h^{-1})	
				$\bar{X}$	SE	$\bar{X}$	SE	$\bar{X}$	SE
6	June 11	15	3	20.6	9.2	32.8	17.9	53.4	27.1
12	June 13	15	3	24.5	0.7	26.8	10.5	51.4	11.2
18	June 13, 14	15	3	40.9	16.1	17.0	16.1	57.9	31.1
24	June 12	15	3	55.7	13.3	21.3	22.8	77.1	16.1
30	June 11, 12	15	3	42.1	20.6	-17.6	32.4	24.5	14.7
36	June 14	15	3	48.5	7.9	-29.2	5.8	19.3	3.5
